# Development of Comprehensive Personal Health Records Integrating Patient-Generated Health Data Directly From Samsung S-Health and Apple Health Apps: Retrospective Cross-Sectional Observational Study

**DOI:** 10.2196/12691

**Published:** 2019-05-28

**Authors:** Se Young Jung, Jeong-Whun Kim, Hee Hwang, Keehyuck Lee, Rong-Min Baek, Ho-Young Lee, Sooyoung Yoo, Wongeun Song, Jong Soo Han

**Affiliations:** 1 Office of eHealth Research and Business Seoul National University Bundang Hospital Seongnam-si Republic of Korea; 2 Department of Family Medicine Seoul National University Bundang Hospital Seongnam-si Republic of Korea; 3 Department of Otorhinolaryngology Head and Neck Surgery Seoul National University Bundang Hospital Seongnam-si Republic of Korea; 4 Department of Pediatrics Seoul National University Bundang Hospital Seongnam-si Republic of Korea; 5 Department of Plastic Surgery Seoul National University Bundang Hospital Seongnam-si Republic of Korea; 6 Department of Nuclear Medicine Seoul National University Bundang Hospital Seongnam-si Republic of Korea

**Keywords:** personal health record, patient generated health data, lifelog, electronic medical record, mobile phone

## Abstract

**Background:**

Patient-generated health data (PGHD), especially lifelog data, are important for managing chronic diseases. Additionally, personal health records (PHRs) have been considered an effective tool to engage patients more actively in the management of their chronic diseases. However, no PHRs currently integrate PGHD directly from Samsung S-Health and Apple Health apps.

**Objective:**

The purposes of this study were (1) to demonstrate the development of an electronic medical record (EMR)–tethered PHR system (Health4U) that integrates lifelog data from Samsung S-Health and Apple Health apps and (2) to explore the factors associated with the use rate of the functions.

**Methods:**

To upgrade conventional EMR-tethered PHRs, a task-force team (TFT) defined the functions necessary for users. After implementing a new system, we enrolled adults aged 19 years and older with prior experience of accessing Health4U in the 7-month period after November 2017, when the service was upgraded.

**Results:**

Of the 17,624 users, 215 (1.22%) integrated daily steps data, 175 (0.99%) integrated weight data, 51 (0.29%) integrated blood sugar data, and 90 (0.51%) integrated blood pressure data. Overall, 61.95% (10,919/17,624) had one or more chronic diseases. For integration of daily steps data, 48.3% (104/215) of patients used the Apple Health app, 43.3% (93/215) used the S-Health app, and 8.4% (18/215) entered data manually. To retrieve medical documentation, 324 (1.84%) users downloaded PDF files and 31 (0.18%) users integrated their medical records into the Samsung S-Health app via the Consolidated-Clinical Document Architecture download function. We found a consistent increase in the odds ratios for PDF downloads among patients with a higher number of chronic diseases. The age groups of ≥60 years and ≥80 years tended to use the download function less frequently than the others.

**Conclusions:**

This is the first study to examine the factors related to integration of lifelog data from Samsung S-Health and Apple Health apps into EMR-tethered PHRs and factors related to the retrieval of medical documents from PHRs. Our findings on the lifelog data integration can be used to design PHRs as a platform to integrate lifelog data in the future.

## Introduction

The increase in the prevalence of preventable chronic diseases has become an enormous problem worldwide in recent decades [1,2]. Chronic diseases impose an immense societal and financial burden [3,4]. Three of every five people with type 2 diabetes develop complications such as stroke, heart disease, and eyesight problems. The rate of avoidable hospitalization for hypertension, which is a major risk factor for heart disease, has increased more than 90% over the last 10 years [5]. The main reason for this increase is that patients tend to neglect chronic conditions, because most of these diseases are indolent during subclinical stages. Therefore, it is important to manage chronic diseases before they transition from the subclinical to the clinical phase [6,7].

Personal health records (PHRs) have been considered an effective tool to engage patients more actively in the management of their chronic diseases [8-10]. However, most provider-based PHRs have been unsuccessful in engaging patients in their health care because most patients and health care professionals have failed to obtain benefits from PHRs [11,12]. The health-related information in PHRs is inconsistent and inaccurate because most administrative functions in PHRs are solely dependent on PHR users [13,14]. If users are not interested in inputting information into PHRs, PHRs become useless. Therefore, patient-generated health data (PGHD) are crucial determinants of the success of PHRs, as doctors can recommend or prescribe treatments based on PGHD in PHRs [15,16]. PGHD are health-related data recorded, created, or gathered by or from patients, caregivers, or family members to monitor and address a health concern [15]. PGHD generally include health history, treatment history, biometric data, symptoms, and lifestyle choices. From these data, biometric and lifelog data can be collected via objective methods such as wearables, smartphones, or smart devices.

Seoul National University Bundang Hospital (SNUBH) has had its own onsite electronic medical record (EMR) solution since 2003. It also started an EMR-tethered PHR system called Health4U in June 2013, integrating PGHD such as daily steps, sleep patterns, weight, and blood pressure directly from Samsung S-Health and Apple Health apps for the first time beginning in November 2017, and was the first hospital in the world to do so [14,17,18]. In particular, daily steps are recorded automatically by the mere use of a smartphone in everyday life, and the information can be streamed into Health4U without much effort. Doctors and nurses can check patients’ daily activities by merely monitoring those records. Health4U also adopted medical document download functions for health information exchange. This study explored the features of EMR-tethered PHRs that can integrate S-Health and Apple Health data and investigated factors related to the use pattern of the lifelog integration and medical document download functions in Health4U.

## Methods

### Development Process

A task-force team (TFT) of 5 attending physicians, 3 nurses, and 4 engineers was formed to upgrade Health4U. In June 2017, the TFT began conducting an analysis of a previous version of Health4U, and for 6 months, they defined functions that should be upgraded based on this analysis (Table 1).

**Figure 1:**
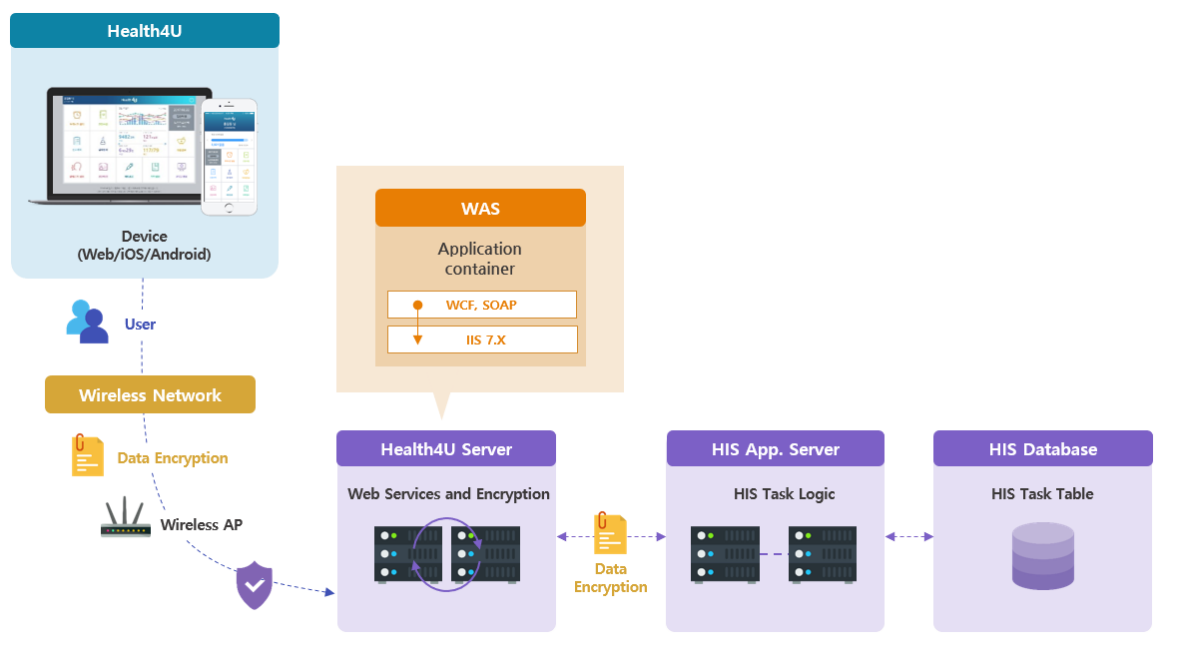
System architecture. HIS: hospital information system. WAS: web application server.

Health4U is installed on a personal computer and mobile. The overall system architecture is described in Figure 1. When a user logs into Health4U, the server communicates through the hospital information system (HIS) server attached to the HIS database. All data streamed between the Health4U server and the HIS app server are encrypted.

**Table 1:** Important functionalities identified by the task-force team.

Domain	Functions
Medical record	View the medical history
Lab results	Change the date standard from the reporting date to the order-issued date
Prescription history	Provide medication information (eg, drug image, usage, effect, and side effects)
Diagnosis history	View function for diagnosis history
Alarm setting for medication	Push notification for taking medicine/note whether to take a dose
Vaccination	View vaccination history (eg, for children and adults)
Health notes	Lifestyle data management for activity, weight, blood pressure, blood sugar test, sleep, and stress Link to Samsung S-Health and Apple Health App data
Medical info	Link to check the encyclopedia (medical information) of the famous web portal called NAVER
Educational material	Provide education animation related to the patient’s diagnosis
Blue button	Download and view the PDF of medical history Transfer to Samsung S-Health in the form of C-CDA^a^ file
View health data	View lifestyle data (eg, activity, weight, blood pressure, and sleep)/push the numerical criteria for recommendation
Login	Fingerprint login
Link to external apps	Link to app for hospital webpage and BESTguide app

^a^C-CDA: Consolidated Clinical Document Architecture.

### Upgrade of the Previous Version of Health4U

**Figure 2:**
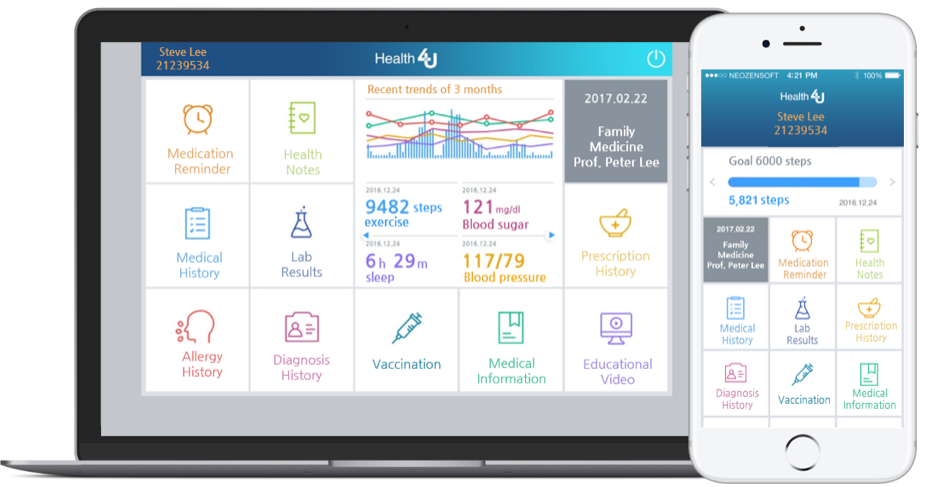
The Health4U app for personal computer and mobile phone.

A previous version of Health4U was developed as an EMR-tethered PHR system based on the needs of users and was composed of five parts: visit history, drug notification, prescription history, laboratory results, and management of a self-administered component (called the Health Notes; Figure 2) [14].

**Figure 3:**
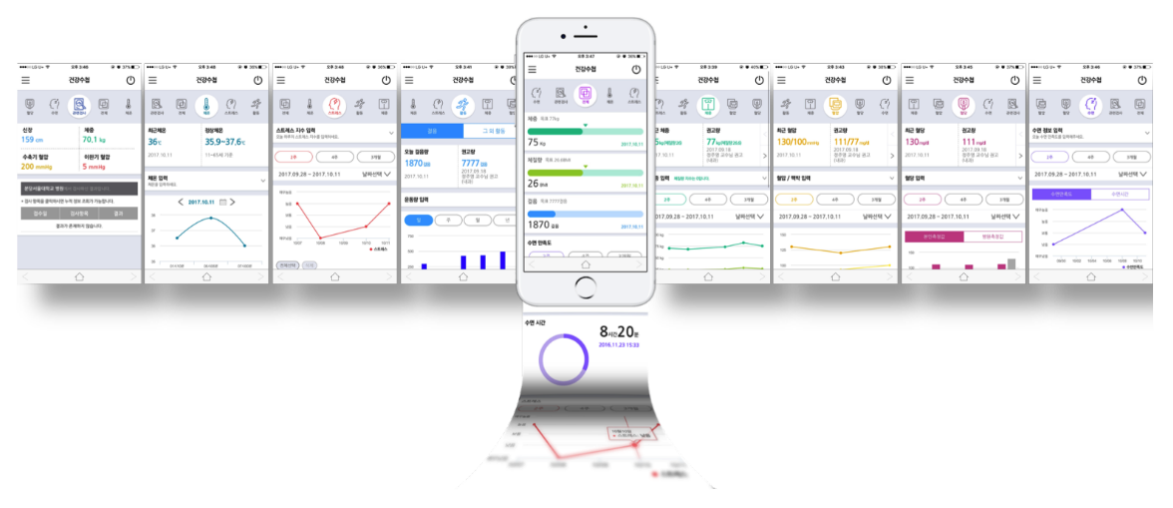
Screenshots of Lifelog management.

The TFT concluded that the integration of PHRs into EMR was not enough to engage patients to manage their diseases, and PGHD integration into both PHRs and EMR can be a key feature that attracts users’ interest. Therefore, we added five new functions to the previous version: tracking of diagnosis history; push notifications for taking medicine; integration of lifelog data including activity, blood pressure, blood sugar, sleep, and stress; fingerprint login; and links to external apps such as an indoor navigation app called BESTguide and a cardiovascular disease management app called Heart4U (Figure 3) [19].

**Figure 4:**
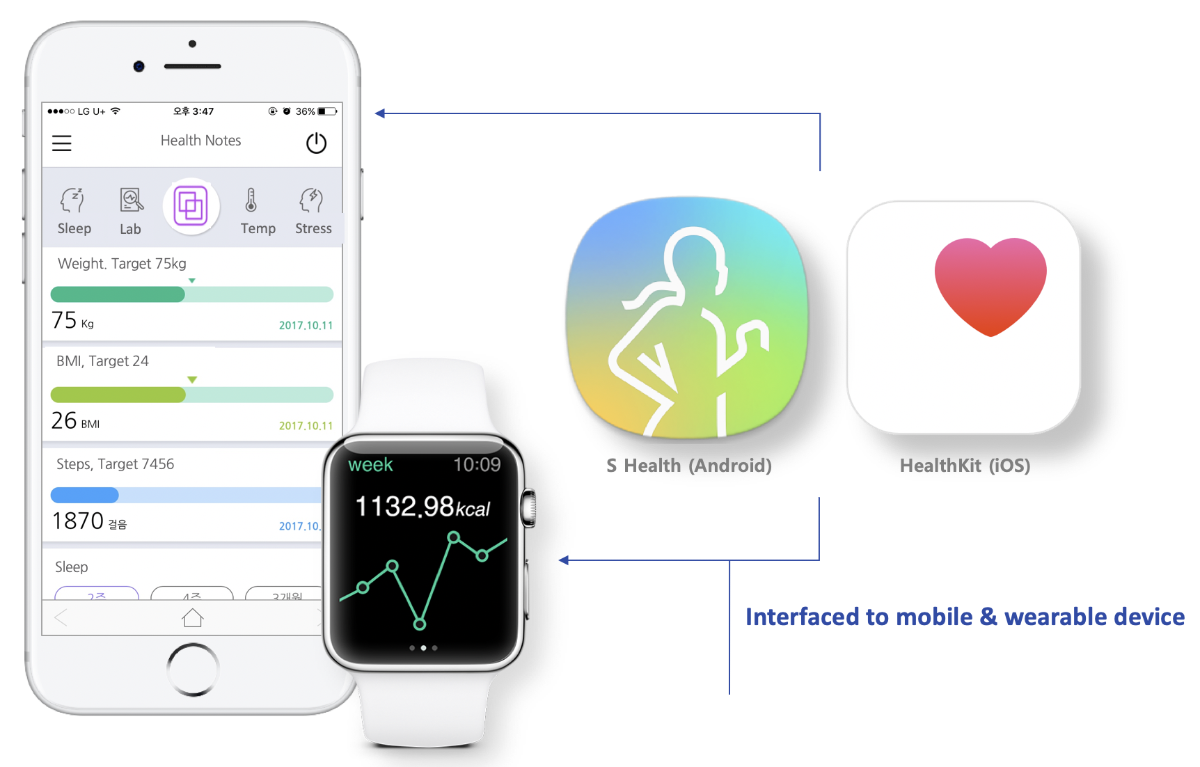
Direct integration of mobile and wearable data.

**Figure 5:**
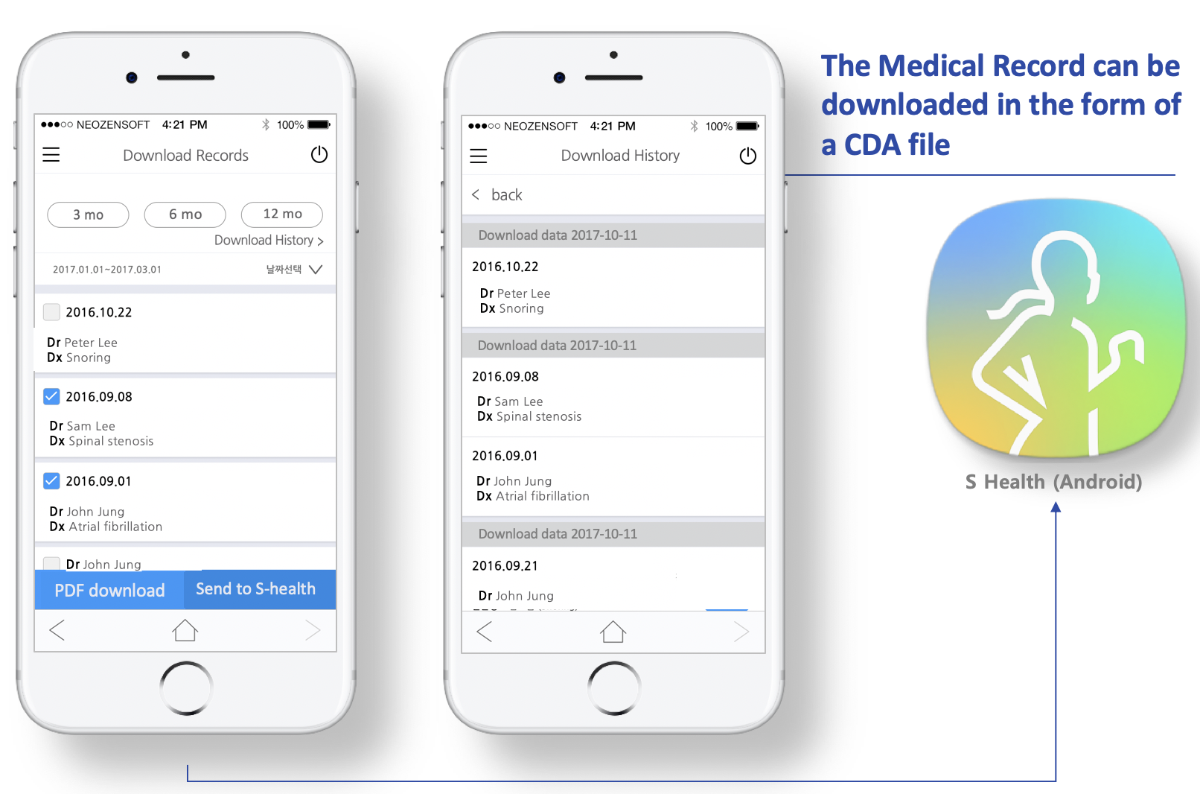
Function for the download of medical records. CDA: clinical document architecture.

**Figure 6:**
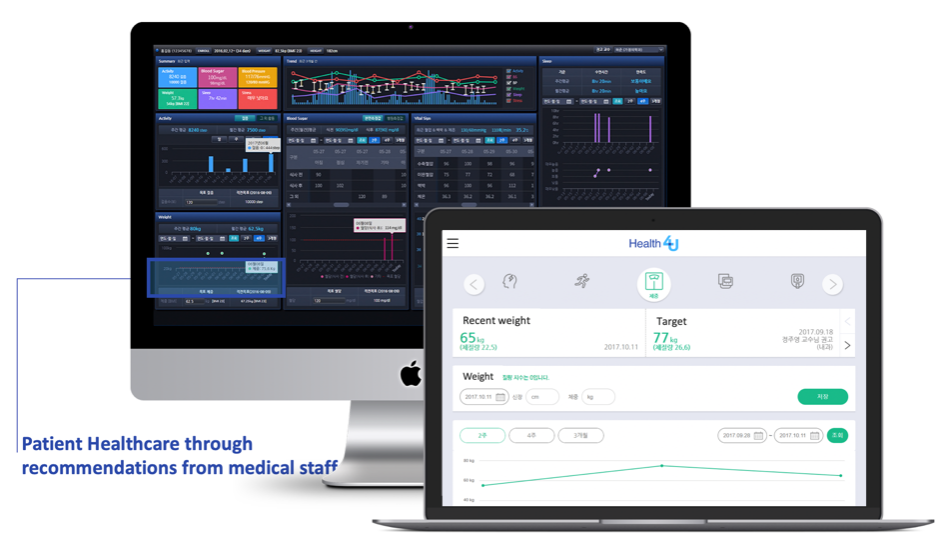
Dashboard for doctors.

In particular, the TFT designed a key function that integrated lifelog data directly from Samsung S-Health and Apple Health apps. This is the first instance worldwide of use these two platforms as portals to collect lifelog data for EMR-tethered PHRs (Figure 4). The TFT believed that patients no longer needed to complete structured questionnaires such as the International Physical Activity Questionnaire for tracking daily activities, and doctors could check patients’ daily steps on the dashboard without much effort if we were successful in implementing the daily steps integration function. Additionally, information in PHRs can be downloaded in PDF format or directly integrated into the Samsung S-Health app via the Consolidated Clinical Document Architecture (C-CDA) file (Figure 5). Furthermore, we developed a dashboard for doctors to see patients’ lifelog records to provide recommendations based on their results (Figure 6).

### Study Population

This study used cross-sectional data extracted from a clinical data warehouse of SNUBH. The enrollees were selected from a sample of adults aged 19 years and older, with prior experience in accessing Health4U in the 7-month period after November 2017, when the service was upgraded. The study participants were selected regardless of prior hospitalization to SNUBH and did not receive any rewards from the research when they downloaded and used the app. A total of 17,624 patients were included in this study.

Activity tracking is important for managing chronic diseases. Therefore, we planned to analyze data on daily steps integration as a surrogate marker of easily obtainable activity data, because steps data are recorded whenever users carry their smartphones or put on their wearables such as smart watches. If patients use a Bluetooth-based scale, sphygmomanometer, or blood sugar checker, the data generated by those devices can be integrated into Health4U through Samsung S-Health and Apple Health apps. Additionally, patients manually input those data when they see the results on their devices. Therefore, we planned to analyze weight, blood pressure, and blood sugar data to explore how patients integrate them into their daily lives. Finally, we planned to evaluate factors associated with the use patterns of the PDF and C-CDA download functions because they can be used to exchange health information via social media, and we wanted to gauge patient interest in those functions.

All analyses were performed using ﻿Stata 14.0 (Stata Corp, College Station, TX). A *P* value<.05 was considered significant. The requirement of informed consent was waived because we used anonymized the data. This research was approved by the Institutional Review Board of Human Research of SNUBH.

## Results

### Principal Results

Among the 17,624 users of Health4U from November 2017 to May 2018, 215 (1.22%) users integrated daily steps data, 175 (0.99%) users integrated weight data, 51 (0.29%) users integrated blood sugar data, and 90 (0.51%) users integrated blood pressure data. Overall, 61.95% (10,919/17,624) had one or more chronic diseases. For the function of retrieving medical documentation, 324 (1.84%) users downloaded PDF files and 31 (0.18%) patients integrated their medical records into Samsung S-Health via the C-CDA download function (Table 2).

**Table 2:** Baseline characteristics (N=17,624).

Variable		n (%)
**Age (years)**		
	19-40	3848 (21.83)
	41-60	7043 (39.96)
	60-80	5710 (32.40)
	≥80	1023 (5.80)
**Gender**		
	Male	9142 (51.87)
	Female	8482 (48.13)
**Having a spouse**		
	Yes	11,713 (79.00)
	No	3113 (21.00)
**Educational level**		
	Elementary school and lower	1497 (10.22)
	Middle school degree	1249 (8.53)
	High school degree	4364 (29.79)
	College degree and higher	7539 (51.46)
**Occupation**		
	Blue collar	1853 (12.49)
	White collar	4522 (30.48)
	Student	405 (2.73)
	Other jobs	4605 (31.04)
	No job	3449 (23.25)
**Patient type**		
	New	1173 (6.66)
	Returning	16,451 (93.34)
**Number of chronic diseases^a^**		
	0	6705 (38.04)
	1	4112 (23.33)
	2	2534 (14.38)
	3	1496 (8.49)
	4	989 (5.61)
	≥5	1788 (10.15)
**Hypertension**		
	Yes	2562 (14.54)
	No	15,062 (85.46)
**Diabetes mellitus**		
	Yes	2267 (12.86)
	No	15,357 (87.14)
**Dyslipidemia**		
	Yes	1843 (10.46)
	No	15,781 (89.54)


**Cancer**		
	Yes	5948 (33.75)
	No	11,676 (66.25)
**Prior hospitalization to SNUBH^b^**		
	Yes	14,834 (84.17)
	No	2790 (15.83)
**Departments**		
	Cancer center	2066 (11.72)
	Internal medicine or family medicine	4074 (23.12)
	Neurovascular center	1566 (8.89)
	Surgery	2491 (14.13)
	Other departments	7427 (42.14)
**Daily steps integration**		
	Yes	215 (1.22)
	No	17,409 (98.78)
**Weight integration**		
	Yes	175 (0.99)
	No	17,449 (99.01)
**Blood sugar integration**		
	Yes	51 (0.29)
	No	17,573 (99.71)
**Blood pressure integration**		
	Yes	90 (0.51)
	No	17,534 (99.49)
**PDF download**		
	Users	324 (1.84)
	Nonusers	17,300 (98.16)
**C-CDA^c^** **download**		
	Users	31 (0.18)
	Nonusers	17,593 (99.82)

^a^Chronic diseases include diabetes mellitus, dyslipidemia, hypertension, and any type of cancer.

^b^SNUBH: Seoul National University Bundang Hospital.

^c^C-CDA: Consolidated Clinical Document Architecture

For platforms integrating daily steps, 104 patients used the Apple Health app (HealthKit), 93 patients used the Samsung S-Health app (S-Health), and 18 patients entered data by themselves. For integrating weight, blood sugar, and blood pressure data, participants more often input data manually than in other ways (Table 3).

Table 4 shows the trends of patients’ recordings in each quarter, who integrated lifelog data into Health4U. After the upgraded version of Health4U was launched in the fourth quarter of 2017, daily steps data were integrated into Health4U from Samsung S-Health and Apple Health apps.

We hypothesized that the rate of lifelog integration into PHRs and the use of the PDF or C-CDA download functions depend on a user’s age, gender, educational level, occupation, and number of chronic diseases. These variables were included as covariates for multivariable logistic regression, and additionally, two other variables (having a spouse and department visited) were included to adjust for other influential factors.

**Table 3:** Platforms for integrating daily steps, weight, blood sugar, and blood pressure data.

Variable		n (%)
**Daily steps integration (n=215)**		
	Samsung S-Health	93 (43.3)
	Apple Health	104 (48.3)
	Manual input	18 (8.4)
**Weight integration (n=175)**		
	Samsung S-Health	23 (13.1)
	Apple Health	4 (2.3)
	Manual input	148 (84.6)
**Blood sugar integration (n=51)**		
	Samsung S-Health	4 (8)
	Apple Health	4 (8)
	Manual input	43 (84)
**Blood pressure integration (n=90)**		
	Samsung S-Health	10 (11)
	Apple Health	18 (20)
	Manual input	62 (69)

**Table 4:** Number of patient recordings from the third quarter of 2017 to the second quarter of 2018. The upgraded version of Health4U was launched in the fourth quarter of 2017.

Variable		n (%)
**Weight integration (n=457)**		
	Third quarter of 2017	110 (24.0)
	Fourth quarter of 2017	91 (19.9)
	First quarter of 2018	121 (26.5)
	Second quarter of 2018	135 (29.6)
**Blood pressure integration (n=230)**			
	Third quarter of 2017	58 (25.2)
	Fourth quarter of 2017	58 (25.2)
	First quarter of 2018	46 (20.0)
	Second quarter of 2018	68 (29.6)
**Blood sugar integration (n=164)**			
	Third quarter of 2017	30 (18.3)
	Fourth quarter of 2017	44 (26.8)
	First quarter of 2018	40 (24.4)
	Second quarter of 2018	50 (30.5)
**Daily steps integration (n=370)**			
	Third quarter of 2017	0 (0)
	Fourth quarter of 2017	69 (18.6)
	First quarter of 2018	135 (36.5)
	Second quarter of 2018	166 (44.9)

### Analysis of the Lifelog Data Integration Traits

The age group of ≥60 years used the daily steps sync function less often than the reference group. Women used the function 70% less often than men, and the difference was statistically significant (*P*<.01). Educational level, occupation, patient type, number of chronic diseases, and department did not influence the use rate (Table 5).

### Analysis of the Traits Related to Medical Information Download

We found a consistent increase in the odds ratios for the number of chronic diseases related to the PDF download function. The age groups of ≥60 years and ≥80 years ﻿had a lower tendency to use this function. Patients without a spouse used the function 61% more often than those with a spouse. White-collar workers tended to use the function 60% more often than blue-collar workers. Users with a college degree and higher education used the function 2.3 times more often than users who finished elementary school only (Table 6).

In contrast, we could not identify any factors that were significantly associated with the use of the C-CDA download function (Table 7).

We also analyzed the association of daily steps sync function with download functions (PDF and C-CDA) and lifelog integration functions (blood pressure, blood sugar, and weight). We found that daily steps sync was strongly related with the other functions (Table 8). Users of the PDF download, C-CDA integration, blood sugar integration, blood pressure integration, and weight integration functions synced their daily steps 7.8 times, 18.76 times, 18.66 times, 4.68 times, and 41.31 times more, respectively, than users who did not use these functions.

**Table 5:** Multivariable analysis of the factors associated with daily steps synced with Health4U.

Variable		Adjusted odds ratio	*P* value
**Age (years)**			
	19-40	1	
	41-60	0.75	.30
	60-80	0.39	.01
	≥80	N/A^a^	N/A
**Gender**			
	Male	1	
	Female	0.30	<.001
**Having a spouse**			
	Yes	1	
	No	0.92	.78
**Educational level**			
	Elementary school and lower	1	
	Middle school degree	N/A	N/A
	High school degree	4.05	.18
	College degree and higher	5.40	.10
**Occupation**			
	Blue collar	1	
	White collar	2.19	.03
	Student	2.57	.15
	Other jobs	1.91	.14
	No job	1.42	.43
**Patient type**			
	New	1	
	Returning	1.32	.59
**Number of chronic diseases^b^**			
	0	1	
	1	1.39	.23
	2	1.37	.36
	3	2.16	.03
	4	1.88	.15
	≥5	1.73	.19
**Departments**			
	Other departments	1	
	Cancer center	0.76	.46
	Internal medicine or family medicine	0.88	.61
	Neurovascular center	0.85	.67
	Surgery	1.40	.24

^a^N/A: not applicable.

^b^Chronic diseases include diabetes mellitus, dyslipidemia, hypertension, and any type of cancer.

**Table 6:** Multivariable analysis of the factors associated with the use of the PDF download function in the Health4U app.

Variable		Adjusted odds ratio	*P* value
**Age (years)**			
	19-40	1	
	41-60	0.75	.15
	60-80	0.41	.001
	≥80	0.21	.006
**Gender**			
	Male	1	
	Female	0.8	.17
**Having a spouse**			
	Yes	1	
	No	1.61	.009
**Educational level**			
	Elementary school and lower	1	
	Middle school degree	0.67	.49
	High school degree	1.46	.37
	﻿College degree and higher	2.31	.046
**Occupation**			
	Blue collar	1	
	White collar	1.60	.049
	Student	2.25	.06
	Other jobs	1.07	.80
	No job	0.71	.26
**Patient type**			
	New	1	
	Returning	0.79	.41
**Number of chronic diseases^a^**			
	0	1	
	1	3.53	<.001
	2	4.18	<.001
	3	4.26	<.001
	4	4.45	<.001
	≥5	6.25	<.001
**Departments**			
	Other departments	1	
	Cancer center	0.77	.29
	Internal medicine or family medicine	0.78	.17
	Neurovascular center	0.79	.40
	Surgery	0.75	.24

^a^Chronic diseases include diabetes mellitus, dyslipidemia, hypertension, and any type of cancer.

**Table 7:** Multivariable analysis of the factors associated with the use of the Consolidated Clinical Document Architecture integration function in the Samsung S-Health app.

Variable		Adjusted odds ratio	*P* value
**Age (years)**			
	19-40	1	
	41-60	3.00	.17
	60-80	1.89	.50
	≥80	N/A^a^	N/A
**Gender**			
	Male	1	
	Female	0.59	.33
**Having a spouse**			
	Yes	1	
	No	2.25	.14
**Educational level**			
	Elementary school and lower	1	
	Middle school degree	0.47	.48
	High school degree	0.78	.45
	College degree and higher	N/A	N/A
**Occupation**			
	Blue collar	1	
	White collar	0.89	.85
	Student	2.48	.50
	Other jobs	0.91	.90
	No job	0.40	.30
**Patient type**			
	New	1	
	Returning	1.12	.91
**Number of chronic diseases^b^**			
	0	1	
	1	1.09	.14
	2	1.04	.05
	3	1.23	.25
	4	1.82	.70
	≥5	1.96	.88
**Departments**			
	Other departments	1	
	Cancer center	0.69	.63
	Internal medicine or family medicine	1.26	.64
	Neurovascular center	0.44	.44
	Surgery	0.31	.27

^﻿^^a^N/A: not applicable.

^b^Chronic diseases include diabetes mellitus, dyslipidemia, hypertension, and any type of cancer.

**Table 8:** Multivariable analysis of the association of daily step sync function with other functions. In this analysis, the outcome variable was daily steps synced.

Variable		Adjusted odds ratio	*P* value
**Age (years)**			
	19-40	1	
	41-60	0.62	.12
	60-80	0.16	<.001
	≥80	N/A^a^	N/A
**Gender**			
	Male	1	
	Female	0.36	.001
**Having a spouse**			
	Yes	1	
	No	0.67	.27
**Educational level**			
	Elementary school and lower	1	
	Middle school degree	N/A	N/A
	High school degree	4.79	.22
	College degree and higher	7.45	.12
**Occupation**			
	Blue collar	1	
	White collar	2.07	.097
	Student	2.52	.24
	Other jobs	1.93	.20
	No job	1.58	.43
**Patient type**			
	New	1	
	Returning	1.41	.59
**Departments**			
	Other departments	1	
	Cancer center	0.68	.37
	Internal medicine or family medicine	0.64	.18
	Neurovascular center	0.51	.18
	Surgery	0.87	.68
**PDF download**			
	No	1	
	Yes	7.80	<.001
**C-CDA^b^ download**			
	No	1	
	Yes	18.76	<.001
**Blood pressure integration**			
	No	1	
	Yes	18.66	<.001
			
**Blood sugar integration**			
	No	1	
	Yes	4.68	.02
**Weigh integration**			
	No	1	
	Yes	41.31	<.001

^a^N/A: not applicable.

^b^C-CDA: Consolidated Clinical Document Architecture

## Discussion

### Overview

To the best of our knowledge, this is be the first study to link mobile phone-based health care platforms such as Samsung S-Health and Apple Health to EMR-tethered PHRs to collect lifelog data. We hypothesized that there can be large hurdles despite our efforts to integrate lifelog data directly into PHRs from the Samsung S-Health and Apple Health apps. We also hypothesized that the adoption rate of new functions, such as lifelog integration and medical document downloads, depends on a user’s age, gender, occupation, educational status, and number of chronic conditions. We analyzed the results of daily steps integration as a representative marker of lifelog data because it can be generated easily from wearables and smartphones and integrated effortlessly into PHRs.

We found that the use rate of lifelog integration is very low, given the total number of registered users during the study period: 1.22% for daily steps, 0.99% for weight, 0.29% for blood sugar, and 0.51% for blood pressure (Table 2). However, we already presented a hypothesis based on the results of previous research that the use rate of the self-administration function in EMR-tethered PHRs can be lowered if patients have difficulties inputting data into PHRs [14]. A previous study also revealed that the successful adoption of PHRs depends on the patient-clinician relationship and the promotion of the technology to physicians [8]. If we promote the use of self-administered functions that can be integrated automatically from smartphones among patients, the results might be different. Although the use rate for lifelog integration was low, we found interesting results. Patients who integrated daily steps used the Samsung S-Health and Apple Health apps more than the self-administration function. Compared to daily steps integration, weight, blood sugar, and blood pressure data were integrated by patients who input their data manually. If users are registered to access their PHRs and launch the Health4U app, data on their daily steps are automatically transferred from their smartphones to Health4U, thus making it unnecessary for users to remember to integrate their activity data into Health4U. We concluded that the difference between the integration of daily steps and that of other lifelog data is attributable to the different methods by which patient data are collected in PHRs. We proved in a previous study that health outcomes can be improved if we integrate lifelog data into EMR-tethered PHRs in order to enable doctors to provide recommendations based on shared lifelog records [16]. However, we also found that patients usually do not want to put on wearables, such as smart watches, for long periods of time. Therefore, collecting activity data from only smartphones and adjusting these data with possible real-world activity data can be a good option for patients and health care professionals to obtain continuous activity data.

We also found that daily steps integration was associated with gender and the age group of ≥60 years. Women and people aged ≥60 years were less likely to integrate their daily steps data. These results are similar to those of previous studies. Jung et al [14] found that women are less likely than men to use self-administered functions in EMR-tethered PHRs [14]. Studies found that men were more likely to consider computer use enjoyable, be confident about using the internet and PHRs, and be engaged users of PHRs as compared to women [20,21]. We were unable to find consistent results for age groups with statistical significance, but the odds ratios were consistently decreased when patient groups were older: The group of adults aged ≥60 years was 61% less likely to integrate daily steps data into PHRs. To integrate daily steps into the Health4U app from the Samsung S-Health or Apple Health app, users must launch the S-Health or Apple Health app at least once. It is possible that the elderly are not interested in health-related functions installed on their smartphones. As shown above, daily steps can be integrated more easily from the S-Health and Apple Health apps than from self-administration; the age barrier for lifelog integration can be addressed by encouraging patients to use health-related preinstalled apps on their smartphones.

Finally, we found that the more chronic diseases a patient has, the more frequently the patient uses the PDF download function. To our knowledge, this is the first study to show that the PDF download function for medical information in EMR-tethered PHRs is associated with the number of chronic conditions. This finding suggests that patients with multiple chronic diseases want to maintain their medical records independently. In 2015, the Office of the National Coordinator for Health IT issued a paper [22] wherein they proposed that future PHRs should be based on HIE because a PHR tethered to a single institution is not sufficient for managing diseases, as patients usually visit multiple hospitals and institutions. Another study showed that willingness to enroll in PHRs was associated with the presence of doctor-diagnosed chronic diseases [23]. One other study found that HIE can decrease the number of future encounters and future readmissions among patients with chronic diseases [24]. Our findings, in conjunction with the results of these previous studies, suggest that patients with multiple chronic diseases are already aware of the importance of the HIE and PHR functions for multiple chronic conditions, and these functions may help improve health outcomes, if implemented appropriately. For example, patients may become interested in systemically summarized notes about their chronic health conditions.

Participants in the age groups of ≥60 years and ≥80 years ﻿had a lower tendency to use the PDF download function. The result of the lower use rate among these older age groups is similar to that of the lower use rate of daily steps integration, indicating that older age can be a hurdle to using advanced PHR functions. A previous study revealed that ﻿usability concerns are barriers to PHR adoption and use [25].﻿ Other research proved that older people are likely to have lower literacy for new technology compared to younger people [26]. Therefore, to increase the use rate of lifelog integration functions, we must consider usability and technology literacy issues. White-collar workers tend to use the function 60% more often than blue-collar workers. White-collar workers today primarily perform their jobs on computers and portable devices [27], which may account for the difference in these use rates. Users with college degrees and higher education used the function 2.3 times more often than users who finished only elementary school. Use rates may also be related to technology literacy issues. To promote the use of PHR functions for HIE, we must consider the use of advertisements targeted toward groups with different educational levels and occupational backgrounds.

### Limitations

﻿This research is a single-center retrospective cross-sectional study, which makes it difficult to identify causal relationships, and the study lacks accurate information on improvement in the health outcomes of PHR users who integrated lifelog data. However, this study found characteristics of users related to lifelog integration and medication documentation downloads. Based on these findings, future studies can be performed effectively.

There are limitations to generalizing the results of this study because it involved only one tertiary hospital and the participation rate was low. However, because this study focused on the use of novel functions implemented in EMR-tethered PHRs at a large hospital, the results will serve as an important background for medical institutions intending to develop similar features or for national agencies planning to develop HIE-based PHRs.

### Conclusions

This is the first study to identify factors related to the integration of daily steps from Samsung S-Health and Apple Health apps into EMR-tethered PHRs and the factors related to the retrieval function of medical documents from PHRs. The finding that patients with more chronic diseases tend to download their medical information more frequently can serve as the basis for enhancement of the features of an EMR-tethered PHR system for HIE. Additionally, findings on lifelog data integration can be used to design PHRs as a platform to integrate lifelog data in the future.

## Abbreviations

C-CDA: Consolidated Clinical Document Architecture
EMR: electronic medical record
HIS: hospital information system
PGHD: Patient-generated health data
PHR: personal health record
SNUBH: Seoul National University Bundang Hospital
TFT: task-force team
